# Cross-cultural adaptation and psychometric validation of the brief assessment of recovery capital (BARC-10) scale into Bangla

**DOI:** 10.1097/MD.0000000000035882

**Published:** 2024-01-12

**Authors:** Md. Tanvir Rahman Shah, Mohsin Ali Shah, Md. Rashid- Al-Mahmood, Kamrun Nahar, Md. Sazib Miah, Taslima Yasmeen Chowdhury

**Affiliations:** a Psychiatry, Bangabandhu Sheikh Mujib Medical University (BSMMU), Shahbag, Dhaka, Bangladesh; b Rangpur Medical College, Rangpur, Bangladesh; c Department of Psychiatry, Bangabandhu Sheikh Mujib Medical University (BSMMU), Shahbag, Dhaka, Bangladesh; d Physical Medicine and Rehabilitation, Bangabandhu Sheikh Mujib Medical University (BSMMU), Shahbag, Dhaka, Bangladesh; e Northern International Medical College, Dhaka, Bangladesh; f Abdul Malek Ukil Medical College, Noakhali, Bangladesh; g Mymensingh Medical College, Mymensingh, Bangladesh; h International Organization for Migration, Cox’s Bazar, Bangladesh.

**Keywords:** deaddiction, recovery capital Bangla version, substance used disorder

## Abstract

Recovery capital is a construct central to the substance use disorder treatment and recovery field. Lack of structured instrument for its assessment in the local context necessitated the translation of the English self-assessment version of the “Brief Assessment of Recovery Capital” (BARC-10) scale to Bangla and the study of its psychometric properties. The objective was to develop a culturally adapted and validated Bangla version of the BARC-10 scale for substance use disorders patients. This study conducted in the period of January 2021 to March 2022 in the department of Psychiatry of a tertiary hospital and central drug addiction treatment center. Initially BARC-10 questionnaire was translated into Bangla (T1 and T2) by 2 separate translators and then synthesis of a single version (T12) was done based on the previous translations. After that 2 back translations (BT1 and BT2) were done by 2 more translators based on the synthesized version (T12). By reviewing all these forward and backward translations, an expert committee made the pre-final version after making some linguistic modification. Then data collection was done among 100 subjects who were selected purposively. Reliability was assessed by Cronbach alpha. Content validity, face validity and Construct validity by factor analysis were measured. Internal consistency measured by Cronbach alpha found was 0.846. No significant change in Cronbach alpha was observed following deleting any item. Confirmatory factor analysis revealed a good fit to data by a chi-square/df value1.33, RMSEA value 0.058. Kaiser-Meyer-Olkin value (.840) showed sampling adequacy. Exploratory factor analysis of the principal component identified 2 factors which had eigenvalues of more than 1. Scree plot also revealed similar factors. These 2 factors together explained 53.1% of the variance. All items were loaded under 2 factors after varimax rotation. The validated Bangla version of the BARC-10 demonstrated high internal reliability and validity. It can potentially be applied in “recovery-oriented” deaddiction service.

## 1. Introduction

Substance use disorder (SUD) is a global burden and new challenge to solve for majority of the countries. According to World Health Organization estimates in the year 2016, there was around 2 billion alcohol users and 185 million drug users worldwide.^[1]^ Although the history of SUD is very old in human being, it has become a serious national and global problem in recent times. It not only affects psychological and body functions but also damages economic, individual, family and social factors^[2]^

“Recovery” from addiction increased also. In the US, the Betty Ford Institute Consensus Panel (2007) defined recovery as “a voluntarily maintained lifestyle characterized by sobriety, personal health and citizenship”.^[3]^ Subsequently, the UK Drug Policy Commission (2008) followed up this statement with a definition of recovery as “voluntarily sustained control over substance use which maximizes health and wellbeing and participation in the rights, roles and responsibilities of society”. Both of these definitions involve 3 primary component parts – wellbeing and quality of life, some measure of community engagement or citizenship, and some measure of sobriety.^[4]^ Since the concept of “recovery” is unique in different sociocultural settings, hence an individualized objective instrument suited to the local context is required^[1]^

Researchers and clinicians have devised the construct of “recovery capital” to refer to the sum of resources necessary to initiate and sustain recovery from substance use.^[5]^

The brief assessment of recovery capital (BARC) was operationalized in a 10-item measure. The brief assessment of recovery capital scale (BARC-10) is a strength-based measure that is completed via self-report to assess the level of extended personal, social, physical, and professional resources in an individual’s environment that are used to initiate and sustain recovery including structural supports such as a recovery-supportive living space and community relationships. It is a brief, easy to use as a self and physician administered tool. Not only physicians, other health care providers like the community health workers can also use this tool to assess recovery capital. The 10-item measure of recovery capital captured item representation from all 10 original subscales, was invariant across participant’s geographic locality, gender, had high internal consistency (α = .90), and concurrent validity with the original measure (rpb = .90). As such, the briefer BARC-10 may serve as a useful additional tool for researchers to explain how individuals achieve recovery, and clinicians may find the BARC-10 helpful in establishing care plans and ranking priorities in ongoing client support. There is additional utility in settings where brevity is valued such as health care systems, electronic health records, as well as peer-to-peer recovery support services^[6]^

SUD affecting all age groups with a considerable impact on quality of life. For better understanding the potential of an individual to undergo “recovery” in a manner suited to the local context, the BARC-10 scale needed to be translated to native language, and subsequently, validation needs to be done. Till date there are no validated tools that can be used among Bangladeshi people to measure recovery capital of SUD patients. Moreover, Bangla is also used in some other countries or province in south East Asian area.

Hence, the aim of this study was to develop a Bangla version of the English BARC-10 scale. In this regard, the objectives were an initial translation as per standard procedures and its applications on persons.

The purpose of this study is to examine the psychometric properties of the BARC-10 Bangla among population. In particular, we investigated the reliability (internal consistency), the face and content validity, and construct validity by factor analysis [both confirmatory factor analysis (CFA) and exploratoy factor analysis (EFA)].

## 2. Methodology

It was a cross sectional validation study which was conducted within the period of January 2021 to March 2022, in Outpatient Department and Deaddiction clinic of Department of Psychiatry, of a tertiary hospital of Bangladesh and Central Drug Addiction Treatment Center situated in the capital.

Recovery capital is a construct central to the substance use disorder treatment and recovery field and the BARC was operationalized in a 10-item measure. Questions are on a 6-point scale. One = Strongly Disagree, 2 = Disagree, 3 = Somewhat Disagree, 4 = Somewhat Agree, 5 = Agree, 6 = Strongly Agree. Total scores can range from a minimum of 10 to a maximum of 60. The original form of questions was^[6]^ -

1. There are more important things to me in life than using substances.
2. In general, I am happy with my life.
3. I have enough energy to complete the tasks I set myself.
4. I am proud of the community I live in and feel part of it.
5. I get lots of support from friends.
6. I regard my life as challenging and fulfilling without the need for using drugs or alcohol.
7. My living space has helped to drive my recovery journey.
8. I take full responsibility for my actions.
9. I am happy dealing with a range of professional people.
10. I am making good progress on my recovery journey.

Permission was obtained from original author to translate the BARC-10 Scale into Bangla.

## 3. Research design

### 3.1. Cross-cultural adaptation

#### 3.1.1. Stage I (translation).

BARC-10 questionnaire was translated into Bangla by 2 persons having competency on both Bangla and English. Conceptual translation was done more than literal translation. Jargons were omitted. These were numbered as below-

1. T1: by a psychiatrist (informed translator).
2. T2: by a person who had no medical or clinical background (uninformed translator).

#### 3.1.2. Stage II (synthesis).

First author synthesized 2 translations into 1 translation (T12). Any discrepancies of the translator’s reports were resolved.

#### 3.1.3. Stage III (back translation).

A back translation then was done by 2 independent, bilingual (English and Bangla) translator who were unaware of the original English version. They were numbered as BT1 and BT2.

#### 3.1.4. Stage IV (expert committee review).

An expert bilingual committee consisting of 4 psychiatrists, a clinical psychologist and a language professional explored the semantic, idiomatic, and conceptual equivalence of the items, reviewed them and reached a consensus on discrepancies. Then, pre-final version was produced after the reconciliation report was compiled by the expert committee.

#### 3.1.5. Stage V (pretesting).

The initial form of the pre-final version was given to a group of 10 bilingual (both Bangla and English) patients with substance use disorder from the Outpatient Department. It was administered face-to-face and the participants were asked whether they fully understood all items and whether they had problems with the formulation of the questions and/or answers. This group of participants had the same characteristics with the sample of the study. Researchers reviewed the results with the aim of identifying any modification necessary to improve the Bangla form. From this procedure, some minor revisions (i.e., grammatical, syntax changes on difficulty in completing the scale and understanding the text) were made.

#### 3.1.6. Stage VI.

The final stage in the adaptation process was a submission of all the reports and forms to the expert committee again. In effect it is a process of audit, with all the steps followed and necessary reports followed. Thus, the previously formulated questionnaire which was finalized on expert committee review was considered as final version. The resulting instrument adapted for Bangla speaking population was named as BARC-10 Scale Bangla.

### 3.2. Procedure of the data collection

Subjects of study were patients of 18 to 60 year, who were suffering from SUD; diagnosed by psychiatrists as per The Diagnostic and Statistical Manual Of Mental Disorders V criteria. They were able to “read” and “understand” Bangla. SUD subjects who had severe mental illness; serious medical comorbidity; moderate or severe withdrawal/intoxication on any substance as per clinical assessment; or who were mute, noncommunicable, and/or unwilling to participate in the study were excluded from the research.

In this study, sample size was calculated based on item and sample ratio, 1:10. As the total item number was 10, total 100 sample was considered. Purposive sampling was done.

Informed and understood written consent were taken from every patient before enrollment. The procedure was taken approximately 10 minutes for each patient.

Information was collected in 2 separate forms. First 1 was semi-structured questionnaire; which was designed by researcher containing socio-demographic variables such as name, age, sex, occupation, religion, habitant, education, family history of substance use, name of the substances etc. Another form contained the Bangla version of BARC-10. Data were collected through this questionnaire from the patients by face-to-face interview.

### 3.3. Data analysis

After collecting the data it was checked and rechecked for omission, inconsistencies and improbabilities. Data analysis was performed by statistical package for social science IBM (SPSS), version- 23 (IBM corporation, Armonk, NY). Data was strictly monitored, preserved, not to be manipulated by others in different stages of the study. *P* value < .05 was considered significant. Kaiser-Meyer-Olkin (KMO) values between 0.8 and 1 indicate the sampling is adequate.^[7]^

### 3.4. Assessment of validity and reliability of The BARC-10

#### 3.4.1. Reliability.

To measures the internal consistency Cronbach alpha coefficient was used. Researcher assessed Cronbach alpha to find out internal consistency. Intraclass correlation coefficient (ICC) was also obtained.

#### 3.4.2. Validity.

Face validity: 4 members experts committee (Psychiatrists having competency on both Bangla and English) reviewed the Bangla version of BARC-10 and provided their valuable opinion about the face validity.

##### 3.4.2.1. Content validity

Was assessed and validated by expert committee.

##### 3.4.2.2. Construct validity

Researcher assessed construct validity by CFA and EFA.

### 3.5. Ethical consideration

The research protocol was approved by the IRB (Institutional Review Board) of Bangabandhu Sheikh Mujib Medical University, Dhaka on October 09, 2021 NO- Bangabandhu Sheikh Mujib Medical University/2021/9251 in its IRB meeting held on October 02, 2021. In this study precaution was taken to protect confidentiality of the participants. Information identifying the participant was kept to a minimum. There was no physical, psychological, and social risk to the patients. Privacy, anonymity, and confidentiality of data information identifying any patient were maintained strictly. Each patient enjoyed every right to participate or refuse or even withdraw from the study at any point of time. The study conforms to code of ethics of the world medical association (Helsinki Declaration).

## 4. Result

Table 1 showed, mean age of the study subjects was 32.56 ± 9.20 years with a range 18 to 50 years. Most of the study subjects lived in urban area. Maximum study subjects were business person (34.0%) followed by unemployed (32.0%), Service holder (18.0%), student (14.0%) and laborer (2.0%). Almost half of the study subjects were married and 45.0% were unmarried. Maximum study subjects had nuclear family and 35.0% were attached with joint family. Majority of the study subjects had monthly family income > Tk. 30,000. From Table 2 it is found, in 19.0 percent of cases, family member was a drug user. The majority of the study participants used multiple drugs. Tobacco (91.0 %), cannabis (43.0 %), opioids (36.0 %), and amphetamines (31.0%) were the most commonly used drugs. Forty six point zero percentage of the study participants spent more than Tk. 5000. Ten percentage of the study subjects began using drugs at the age of 10 or below ten, and the vast majority began using drugs between the ages of eleven and twenty. The next table represents results for reliability testing. The item means, the item variances, the item-total correlations, internal consistency coefficient, ICC with 95% confidence interval were summarized in Table 3. Table 4 reveals, model Chi-square value was 34.81, Chi-square/df was 1.33, RMSEA value is < 0.1 (.058). Table 5 shows, the value of KMO (.840) and significance of Bartlett Test of sphericity was below 0.05 ( < .0001). Table 6 shows the communalities between items obtained from factor analysis by principal component which ranges from 0.262 to 0.755. Eigen value is > 1 among 2 factors and these 2-component explained 53% of the variance. Table 7 represents component matrix of the questionnaire after varimax rotation. Eight, out of 10 items were loaded to factor 1 while item 3 and 8 were loaded in factor 2.

**Table 1:** Socio-demographic characteristics of the study subject (N = 100).

	Frequency (n)	Percentage (%)
Residence		
Urban	89	89.0
Rural	11	11.0
Occupation		
Unemployed	32	32.0
Student	14	14.0
Laborer	2	2.0
Service holder	18	18.0
Business	34	34.0
Marital status		
Married	49	49.0
Separated	2	2.0
Divorced	1	1.0
Widower	3	3.0
Unmarried	45	45.0
Type of family		
Nuclear	65	65.0
Joint	35	35.0
Monthly family income (Taka)		
10,000–30,000	28	28.0
>30,000	72	72.0
Age (yr) (Mean ± SD)	32.56 ± 9.20 (18–52)	

**Table 2:** Drug related information (N = 100).

	Frequency (n)	Percentage (%)
Drug abuser in family		
Yes	19	19.0
No	81	81.0
Number of drugs taken		
Single	13	13.0
Multiple	87	87.0
Type of drugs		
Alcohol	15	15.0
Cocaine	1	1.0
Cannabis	43	43.0
Hallucinogens	14	14.0
Opioids	36	36.0
Sedative-hypnotics	7	7.0
Amphetamines	31	31.0
Tobacco	91	91.0
Others	2	2.0
Drug started at age (yr)		
≤10	10	10.0
11–20	81	81.0
>20	9	9.0

**Table 3:** Item characteristics with Item deletion and internal consistency.

Item	Mean	SD	Scale mean if item deleted	Scale variance if Item deleted	Corrected item-total scale correlation	Cronbach alpha if Item deleted
1	5.08	1.134	38.16	71.509	.576	.823
2	3.83	1.658	39.41	64.103	.640	.814
3	4.15	1.486	39.09	66.951	.603	.818
4	4.22	1.580	39.02	66.686	.568	.822
5	3.14	1.676	40.10	69.606	.409	.840
6	4.27	1.362	38.97	68.999	.574	.822
7	3.90	1.720	39.34	66.045	.531	.827
8	4.70	1.219	38.54	75.544	.322	.842
9	5.08	1.152	38.16	71.146	.585	.822
10	4.87	1.134	38.37	70.599	.627	.819
Internal consistency of total Cronbach alpha						.846
Intraclass correlation coefficient (ICC):					0.840 (95% CI .079 ≤ ICC ≤.088)	

**Table 4:** Fit indicators on confirmatory factor analysis of BARC-10 Bangla.

BARC- 10 Bangla	Chi-square	Df	*P* value	Chi-square/df	RMSEA
Full sample	34.81	26	.116	1.33	0.058

BARC-10 = brief assessment of recovery capital scale, Df = Degrees of freedom, RMSEA = Root mean square error of approximation.

**Table 5:** Sampling adequacy test of BARC- 10 Bangla scale.

Kaiser-Meyer-Olkin measure of sampling adequacy		0.840
Bartlett Test of Sphericity	Approx. Chi-square	320.248
	DF	45
	*P* value	<.0001

**Table 6:** Communalities and Eigen values of BARC-10 Bangla scale.

Component	Initial	Extraction	Initial eigenvalues			After varimax rotation	
			Total	% of variance	Cumulative %	% of variance	Cumulative %
1	1.000	.515	4.280	42.799	42.799	36.487	36.487
2	1.000	.545	1.030	10.303	53.102	16.615	53.102
3	1.000	.719	.946	9.457	62.559		
4	1.000	.443	.865	8.650	71.209		
5	1.000	.262	.669	6.691	77.900		
6	1.000	.518	.640	6.397	84.297		
7	1.000	.464	.470	4.702	88.999		
8	1.000	.755	.427	4.274	93.273		
9	1.000	.479	.392	3.916	97.190		
10	1.000	.611	.281	2.810	100.000		

BARC-10 = brief assessment of recovery capital scale.

**Table 7:** Factor pattern after Varimax rotation.

Component	Factor 1	Factor2
1	0.707	0.124
2	0.671	0.308
3	0.411	0.742
4	0.573	0.337
5	0.489	0.148
6	0.705	0.144
7	0.678	0.068
8	0.029	0.869
9	0.634	0.278
10	0.776	0.093

## 5. Discussion

Scientific knowledge must be based on facts which are measurable, testable and reproducible. Today psychometrics plays an important role in psychiatry, public health and even in marketing.^[8]^ The BARC-10 Scale was developed by Corrie L. Vilsaint (2017) the purpose of the study was to adapt and validate the BARC-10 Scale inventory in Bangla. In this study firstly, the English questionnaire was translated into Bangla (T1 and T2) by 2 separate translators and then synthesis of a single version (T12) was done based on the previous translations. After that 2 back translations (BT1 and BT2) were done by 2 more translators based on the synthesized version (T12). By reviewing all these forward and backward translations, synthesized and original version, the expert committee made the pre-final version after making some linguistic modification. Then after pretesting the final questionnaire was ready for the data collection.

All participants were male in this study. Prevalence of substance abuse was more in male in Bangladesh.^[2]^ Some other studies also found male predominance among the addicted respondents most likely due to frustration, failure in academic performance and employment crisis.^[9,10]^ Hindi translated version of BARC scale also took all male subjects in their study.^[1]^

Mean age of the subjects of current study is 32.56 ± 9.20 years which is similar with the study conducted in India.^[1]^ Majority of the respondents were above 20 years in this study. Hasam and Mushahid, 2017 also found 64.6% of the respondents above 20 years in their research among Bangladeshi addicted persons.^[9]^

Eighty nine percentage of the participants of this study were urban dwellers, which supports that substance misuse is an urban problem, in distinction with the healthy village life-style.^[9]^

A significant number of sample (32%) were unemployed, while 14% student, 34% businessman, 18% service holder and 2% laborer in current research. Percentage of unemployed and students were 60 and 25% in the study of Hasam and Mushahid, 2017 while 21.05% and 24.56% in another research.^[9,10]^ This incontinence is probably due to higher mean age and higher academic level (89% at least or above SSC) in present study subjects.

The present research found nearly half of the participants were married, 45% were unmarried and rest were separated, divorced or widowed. Some other studies found higher percentage of addicted individuals were unmarried.^[10]^ Greater number of the subjects belong to nuclear family in this research which supports the study conducted to find prevalence of substance use in Bangladesh^[2]^

“Curiosity” was one of the main reasons to enter in the world of addiction; followed by peer pressure, failure in love, parental neglect and other factors among the present study subjects. Only 19% case there were drug abuser in family. Usually, drug addicts think influence of addicted parents and peer groups were responsible for their starting of substance use.^[9]^

Majority of the subjects of this study were multiple drug user. Among them Tobacco (91.0 %), cannabis (43.0 %), opioids (36.0 %), and amphetamines (31%) were the most commonly used drugs. Alcohol consumption was found among 15% of the subjects. Alcohol was primary substance in the original study. Best et al^[5]^, also found primary substance methamphetamine followed by alcohol and opioid. Socio Economical condition and family income may be a factor for this difference.

Reliability was assessed for the BARC -10 Bangla scale by internal consistency. Cronbach alpha was found 0.846 which was statistically significant. The recommended level of Cronbach alpha is < 0.50 unacceptable, 0.50 to 0.59 poor, 0.60 to 0.69 questionable, 0.70 to 0.79 acceptable, 0.80 to 0.89 good, and ≥ 0.90 excellent. The acceptable ICC level is considered when it is ≥ 0.70.^[8,11]^ Both Cronbach internal consistency coefficient and (ICC) for BARC-10 Bangla scale were above 0.8. Internal consistency was calculated as Cronbach alpha value of 0.86 in the Hindi validation of assessment of recovery capital scale.^[1]^ The study of Development and validation of a BARC-10 for alcohol and drug use disorder found Cronbach alpha value was 0.90.^[6]^ Value of all Cronbach Alpha, if corresponding Item deleted; were less than the total value (0.846). Strength of correlation coefficient were considered as: 0 to 0.2 = negligible, 0.2 to 0.5 = weak, 0.5 to 0.8 = moderate, 0.8 to 1 = strong.^[12]^ Item-total correlation showed for all the items correlation values were above acceptable level of 0.20. Also there was weak positive correlation among question 5 and 8 with total scale while rest of the items had moderate positive correlation. Lowest score was found for Item 8 (0.32); deleting this, would change the Cronbach alpha value to 0.842 from 0.846 which is a negligible difference in value. So, decision was taken to keep that item.

Validity of the BARC-10 Bangla was determined. Face and content validities are regarded as weaker forms of validity.^[8]^ Regarding the face validity, the translation of the instrument seemed to be valid. It was well accepted by the small group of 10 patients as the expert committee had reported.

Content validity can be measured or ensured by ensuring standard back translation process, by literature review and expert panel opinion and by experts with content validity index.^[8]^ Regarding the content validity of this scale, the expert committee opined that the instrument was found to include necessary questions. They assessed every item of the translation by comparing the translations and back translations and reached to the decision.

A CFA was conducted to further examine the factorial validity. Maximum likelihood estimation was used. A number of fit indices were investigated to evaluate the model. We found that chi-square/df value was 1.33, chi squared *P* value was .166, RMSEA value was 0.058. Chi squared *P* value > .05 is acceptable for a good fit while Chi-square/df value in between 1 to 2 denotes food fit. RMSEA is considered very good if it is less or equal to 0.05; good between.05 to .08; moderate between.08 to .1 and unacceptable when > 0.1.^[13]^

To conduct exploratory factor analysis 2 conditions should be met. One is that the KMO coefficient should be more than 0.60 and the significance of Bartlett test for sphericity below 0.0.^[14]^ In this study, the KMO coefficient was 0.840 for the sample and the χ2 value in the Bartlett test was 320.24 (*P* < .0001), indicating that factor analysis could be conducted.

Some criteria can be considered in determining the number of factors rotate: The scree plot test; The eigenvalue-greater-than-1 rule; The percentage for variance accounted for by each component; The percentage of total variance accounted for by the retained components and; The number of interpretable components.^[14]^ EFA of the principal component with varimax rotation was done to detect the factorial structure in observed measurements. It identified 2 components with eigenvalues of more than 1. These 2 components in total explained 53.1% of the variance. Scree plot figure also revealed 2 factors in the construct. The extraction communalities except for item 5 (0.262) are > 0.4. If the communality values low it indicates that the variable has little in common with other variables in the research.

In contrast original BARC -10 scale followed unidimensionality. Samples from Scotland and Australia yielded single linear component that accounted for 59.1% and 54.2% respectively.^[6]^ Therefore, the construct of BARC-10 Bangla has some difference than the original 1.

On varimax rotated component matrix we identified item 3 and 8 correlated most to factor 2 and less to factor 1. Rest of the items correlated mostly to factor 1. We propose Bangla naming of the factors which can be translated as “surrounding factor” (item 1, 2, 4, 5, 6, 7, 9, 10) and “self-responsibility” (item 3,8).

Substance use disorders (SUDs) are chronic illnesses with relapsing-remitting nature. Recovery is a multidimensional process that includes health, quality of life, and many other factors.^[15]^ For alcohol dependence in 5-year follow-up, about 30% to 40% were abstinent and for opioid dependence on methadone maintenance at 5 years up to 25% to 28% were abstinent.^[16]^ It has been seen that recovery in SUDs is an individual’s unique experience and the outcome is variable, the reasons mostly due to different ability of the individual to cope through the journey to recovery. These led Cloud and Granfield to propose the concept of “recovery capital” which draws upon the social and personal resources of the individual to undergo recovery.^[17]^ Recovery capital is a lens that could help identify distinct areas of assets that could be enhanced and obstacles to be addressed in individuals recovery processes.^[18]^ To the researcher’s knowledge, the current study is the first study to culturally adapt and validate the BARC-10 Bangla version. This study employed a systematic scientific method for translation and cultural adaptation that was implemented in several steps followed by several tests of reliability and validity. So, we expect the Bangla version of BARC -10 scale will be an important addition in the field of research among deaddiction and recovery in Bangladesh and people speaking in Bangla. Appendix 1, Supplemental Digital Content, http://links.lww.com/MD/L204.

## 6. Limitations

The study was conducted only in tertiary care hospitals, sample from this institution only may not be representative of overall population of the country. Small sample size may bias the validity of the results. Though requirement of item sample ratio (1:10) has been maintained and acceptable psychometric properties yielded with that sample, larger sample would definitely produce better results. Moreover, test-retest reliability assessment couldn’t be done due to anonymity of the respondents. Criterion validity, concurrent validity, convergent validity and divergent validity could not be assessed due to lack of standard culture specific instruments.

## 7. Conclusion

From the finding of the current study, it can be concluded that the Bangla BARC-10 Scale has sound psychometric properties to use among people with Bangla culture. The study revealed an adequate score of internal consistency measured by Cronbach alpha. Face validity, content validity, construct validity (by CFA and EFA) of Bangla BARC-10 was found satisfactory also. It can be used in the development of recovery-oriented services for SUDs in the future.

**Figure 1.**
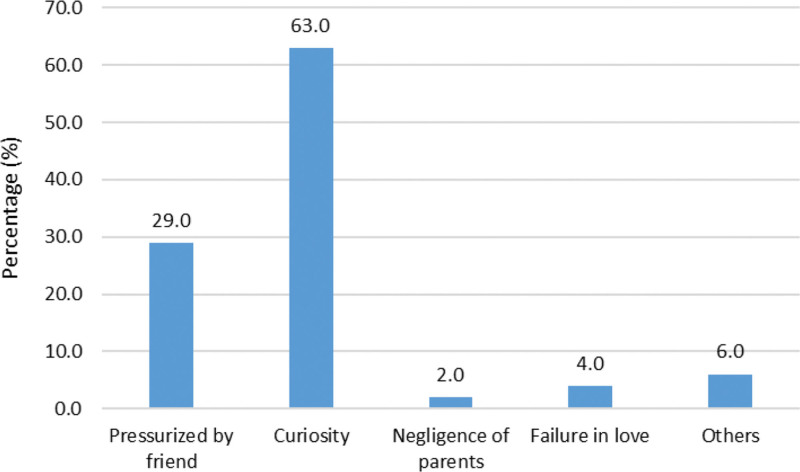
Showing reasons of starting substances. The majority of study subjects began using drugs out of curiosity (63%), followed by peer pressure (29.0 %).

**Figure 2.**
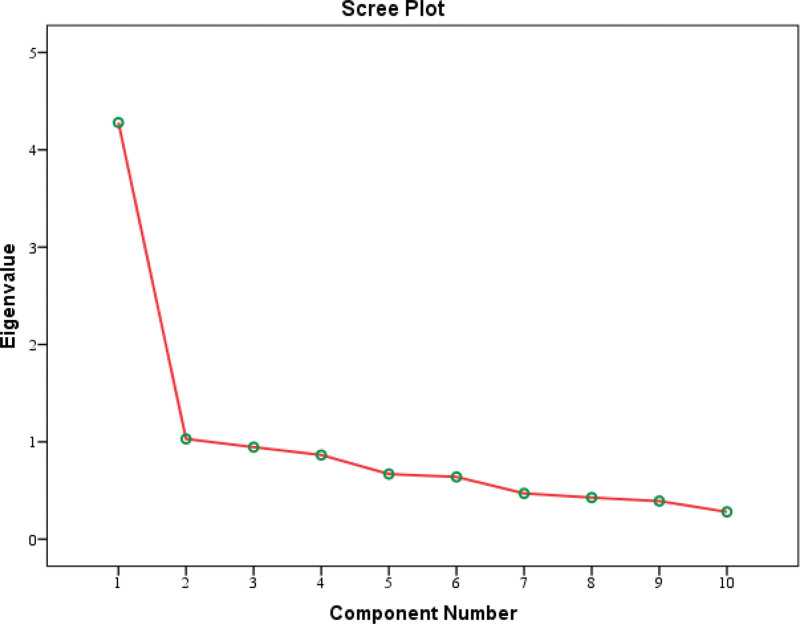
Reveals the scree plot of the questionnaire which signifies the number of factors in the construct. Here the figure reveals two factors in the construct.

## Author contribution

**Conceptualization:** Md. Tanvir Rahman Shah, Mohsin Ali Shah, Md. Rashid- Al-Mahmood, Kamrun Nahar, Md. Sazib Miah, Taslima yasmeen Chowdhury.

**Data curation:** Md. Tanvir Rahman Shah.

**Formal analysis:** Md. Tanvir Rahman Shah, Md. Rashid- Al-Mahmood.

**Methodology:** Md. Tanvir Rahman Shah, Md. Rashid- Al-Mahmood.

**Supervision:** Mohsin Ali Shah.

**Visualization:** Md. Tanvir Rahman Shah.

**Writing – original draft:** Md. Tanvir Rahman Shah, Md. Rashid- Al-Mahmood.

**Writing – review & editing:** Md. Tanvir Rahman Shah, Md. Rashid- Al-Mahmood.

## Supplementary Material
