# Supplementary material for: Cross-cultural adaptation and psychometric validation of the brief assessment of recovery capital (BARC-10) scale into Bangla
Source: Medicine (Baltimore). 2024 Jan 12;103(2):e35882. doi: 10.1097/MD.0000000000035882 (PMC10783311; doi:10.1097/MD.0000000000035882)
Supplement: Supplementary file 1 [file medi-103-e35882-s001.docx]

**Appendix**

T1

| রিকভারি ক্যাপিটাল এর সংক্ষিপ্ত মূল্যাায়ন-১০ (BARC-10 Bangla) | | | | | | |
| --- | --- | --- | --- | --- | --- | --- |
| পরিচিতি নং / নাম - | | | | | তারিখঃ | |
|  | দৃঢ়ভাবে অসম্মত | অসম্মত | কিছুটা অসম্মত | কিছুটা  সম্মত | সম্মত | দৃঢ়ভাবে সম্মত |
| ১। মাদক ব্যবহারের চেয়েও অনেক গুরুতবপূর্ণ বিষয় আমার জীবনে আছে । | ১ | ২ | ৩ | ৪ | ৫ | ৬ |
| .২। সাধারনভাবে আমি আমার জীবন নিয়ে সুখী । | ১ | ২ | ৩ | ৪ | ৫ | ৬ |
| .৩। নিজের জন্য ঠিক করা কাজ শেষ করতে আমি যথেস্ট শক্তি পাই। | ১ | ২ | ৩ | ৪ | ৫ | ৬ |
| ৪। আমি যে সমাজে বাস করি তা নিয়ে গর্বিত এবং নিজেকে এর অংশ বলে অনুভব করি। | ১ | ২ | ৩ | ৪ | ৫ | ৬ |
| ৫। আমি বন্ধুদের কাছ থেকে অনেক সহায়তা পাই। | ১ | ২ | ৩ | ৪ | ৫ | ৬ |
| ৬। মাদকদ্রব্য বা মদ ব্যবহারের প্রয়োজন ছাড়াই আমি আমার জীবনকে চ্যালেঞ্জিং ও পরিপূর্ণ মনে করি । | ১ | ২ | ৩ | ৪ | ৫ | ৬ |
| ৭। আমার বসবাসের জায়গা আমার রিকভারি সফরকে এগিয়ে নিতে সাহায্য করছে । | ১ | ২ | ৩ | ৪ | ৫ | ৬ |
| ৮। আমি আমার কৃতকর্মের পুরো দায়ভার নিই । | ১ | ২ | ৩ | ৪ | ৫ | ৬ |
| ৯। আমি বিভিন্ন ধরনের পেশাজীবীদের সাথে কাজ করতে পেরে খুশি। | ১ | ২ | ৩ | ৪ | ৫ | ৬ |
| ১০। আমার রিকভারি সফরে আমি ভালো উন্নতি করছি । | ১ | ২ | ৩ | ৪ | ৫ | ৬ |
| সর্বমোট স্কোর |  | | | | | |

T 2

| রিকভারি ক্যাপিটাল এর সংক্ষিপ্ত মূল্যাায়ন-১০ (BARC-10 Bangla) | | | | | | |
| --- | --- | --- | --- | --- | --- | --- |
| পরিচিতি নং / নাম - | | | | | তারিখঃ | |
|  | একেবারেই রাজি নই | রাজি নই | কিছুটা রাজি নই | কিছুটা  রাজি | রাজি | পুরোপুরি রাজি |
| ১। নেশা করার চেয়েও অনেক গুরুতবপূর্ণ বিষয় আমার জীবনে আছে । | ১ | ২ | ৩ | ৪ | ৫ | ৬ |
| .২। সাধারনভাবে আমি আমার জীবন নিয়ে সুখী । | ১ | ২ | ৩ | ৪ | ৫ | ৬ |
| .৩। আমি যা করতে চাই, তা শেষ করতে যথেস্ট শক্তি পাই। | ১ | ২ | ৩ | ৪ | ৫ | ৬ |
| ৪। আমি যে সমাজে বাস করি তার অংশ হিসেবে গর্ববোধ করি। | ১ | ২ | ৩ | ৪ | ৫ | ৬ |
| ৫। আমি বন্ধুদের কাছ থেকে অনেক সাহায্য পাই। | ১ | ২ | ৩ | ৪ | ৫ | ৬ |
| ৬। মাদকদ্রব্য বা মদ ব্যবহার করা ছাড়াই আমি আমার জীবনকে উতফুল্ল ও পরিপূর্ণ মনে করি । | ১ | ২ | ৩ | ৪ | ৫ | ৬ |
| ৭। আমি যে সমাজে বাস করি তা আমাকে মাদকাসক্তি থেকে ফিরে আসতে সাহায্য করছে । | ১ | ২ | ৩ | ৪ | ৫ | ৬ |
| ৮। আমি আমার কাজের পুরো দায়ভার নেই । | ১ | ২ | ৩ | ৪ | ৫ | ৬ |
| ৯। আমি বিভিন্ন পেশাজীবী মানুষের সাথে কাজ করতে পেরে খুশি। | ১ | ২ | ৩ | ৪ | ৫ | ৬ |
| ১০। মাদকাসক্তি থেকে ফিরে আসার ক্ষেত্রে আমি ভালো উন্নতি করছি । | ১ | ২ | ৩ | ৪ | ৫ | ৬ |
| সর্বমোট স্কোর |  | | | | | |

T 12

| রিকভারি ক্যাপিটাল এর সংক্ষিপ্ত মূল্যাায়ন-১০ (BARC-10 Bangla) | | | | | | |
| --- | --- | --- | --- | --- | --- | --- |
| পরিচিতি নং / নাম - | | | | | তারিখঃ | |
|  | একেবারেই রাজি নই | রাজি নই | কিছুটা রাজি নই | কিছুটা  রাজি | রাজি | পুরোপুরি রাজি |
| ১। মাদক ব্যবহারের চেয়েও অনেক গুরুতবপূর্ণ বিষয় আমার জীবনে আছে । | ১ | ২ | ৩ | ৪ | ৫ | ৬ |
| .২। সাধারনত আমি আমার জীবন নিয়ে সুখী । | ১ | ২ | ৩ | ৪ | ৫ | ৬ |
| .৩। আমি নিজের কাজ শেষ করতে যথেস্ট শক্তি পাই। | ১ | ২ | ৩ | ৪ | ৫ | ৬ |
| ৪। আমি যে সমাজে বাস করি তার অংশ হিসেবে গর্ববোধ করি। | ১ | ২ | ৩ | ৪ | ৫ | ৬ |
| ৫। আমি বন্ধুদের কাছ থেকে অনেক সহায়তা পাই। | ১ | ২ | ৩ | ৪ | ৫ | ৬ |
| ৬। মাদকদ্রব্য বা মদ ব্যবহারের প্রয়োজন ছাড়াই আমি জীবনের উতফুল্লতা ও পরিপূর্ণতা অনুভব করি । | ১ | ২ | ৩ | ৪ | ৫ | ৬ |
| ৭। আমি যেখানে বাস করি সেটা আমাকে মাদকাসক্তি থেকে ফিরে আসতে সাহায্য করছে । | ১ | ২ | ৩ | ৪ | ৫ | ৬ |
| ৮। আমি যা করি তার পুরো দায়ভার নেই । | ১ | ২ | ৩ | ৪ | ৫ | ৬ |
| ৯। আমি বিভিন্ন পেশার মানুষের সাথে কাজ করতে পেরে খুশি। | ১ | ২ | ৩ | ৪ | ৫ | ৬ |
| ১০। মাদকাসক্তি থেকে ফিরে আসার ক্ষেত্রে আমি ভালো উন্নতি করছি । | ১ | ২ | ৩ | ৪ | ৫ | ৬ |
| সর্বমোট স্কোর |  | | | | | |

BT 1


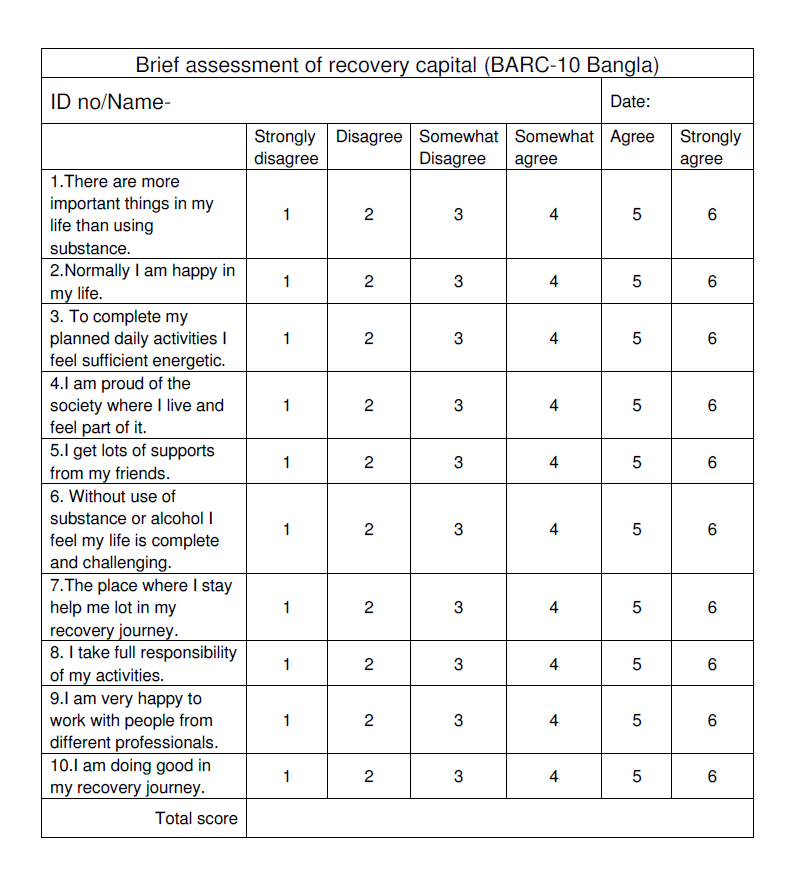


BT 2


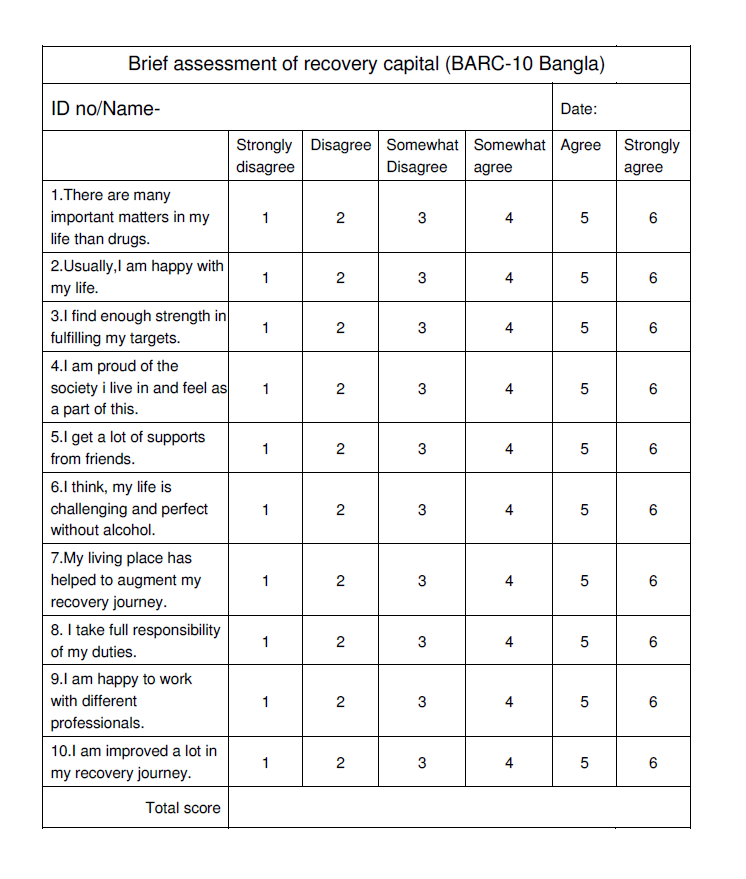


Final Barc 10 bangla

| রিকভারি ক্যাপিটাল এর সংক্ষিপ্ত মূল্যাায়ন-১০ (BARC-10 Bangla) | | | | | | |
| --- | --- | --- | --- | --- | --- | --- |
| পরিচিতি নং / নাম - | | | | | তারিখঃ | |
| ***ক)পারিপার্শ্বিক ভূমিকা*** | | | | | | |
|  | একেবারেই রাজি নই | রাজি নই | কিছুটা রাজি নই | কিছুটা  রাজি | রাজি | পুরোপুরি রাজি |
| ১। মাদক ব্যবহারের চেয়েও অনেক গুরুতবপূর্ণ বিষয় আমার জীবনে আছে । | ১ | ২ | ৩ | ৪ | ৫ | ৬ |
| ২। সাধারনত আমি আমার জীবন নিয়ে সুখী । | ১ | ২ | ৩ | ৪ | ৫ | ৬ |
| ৪। আমি যে সমাজে বাস করি তার অংশ হিসেবে গর্ববোধ করি। | ১ | ২ | ৩ | ৪ | ৫ | ৬ |
| ৫। আমি বন্ধুদের কাছ থেকে অনেক সহায়তা পাই। | ১ | ২ | ৩ | ৪ | ৫ | ৬ |
| ৬। মাদকদ্রব্য বা মদ ব্যবহারের প্রয়োজন ছাড়াই আমি জীবনের উতফুল্লতা ও পরিপূর্ণতা অনুভব করি । | ১ | ২ | ৩ | ৪ | ৫ | ৬ |
| ৭। আমি যেখানে বাস করি সেটা আমাকে মাদকাসক্তি থেকে ফিরে আসতে সাহায্য করছে । | ১ | ২ | ৩ | ৪ | ৫ | ৬ |
| ৯। আমি বিভিন্ন পেশার মানুষের সাথে কাজ করতে পেরে খুশি। | ১ | ২ | ৩ | ৪ | ৫ | ৬ |
| ১০। মাদকাসক্তি থেকে ফিরে আসার ক্ষেত্রে আমি ভালো উন্নতি করছি । | ১ | ২ | ৩ | ৪ | ৫ | ৬ |
| ***খ) স্ব ভূমিকা*** |  |  |  |  |  |  |
| ৩। আমি নিজের কাজ শেষ করতে যথেস্ট শক্তি পাই। | ১ | ২ | ৩ | ৪ | ৫ | ৬ |
| ৮। আমি যা করি তার পুরো দায়ভার নেই । | ১ | ২ | ৩ | ৪ | ৫ | ৬ |
| সর্বমোট স্কোর |  | | | | | |
